# Supplementary material for: Content-rich biological network constructed by mining PubMed abstracts
Source: BMC Bioinformatics. 2004 Oct 8;5:147. doi: 10.1186/1471-2105-5-147 (PMC528731; doi:10.1186/1471-2105-5-147)
Supplement: Additional File 2 — The original results of the above study (non-essential files are deleted to keep the file size under the limit set by BMC bioinformatics). [file 1471-2105-5-147-S2.bz2 › chilibotAdditionalFile2/dip20/293ID7033218E1125/html/NADB_NADA.html]

 


 **NADB** and **NADA** 
  
Found 14 abstracts in PubMed, retrieved 14.  
 

 What does Google say? 
 PDF only 
| .edu only 

---

**Interactive relationship** (e.g. stimulation, inhibition, etc)

**Neutral relationship**- Extracts from certain  **nadB**  mutants complement the extracts prepared from all  **nadA**  mutants for the enzymic synthesis of quinolinate.  Ref: 238844 Eur J Biochem, 1975
- It has been reported that this gene and  **nadA**  are regulated by a positive regulatory protein encoded in the  **nadB**  region.  Ref: 3305482 J Bacteriol, 1987
- In several bacteria, the gene in the position occupied by TM1643 encodes an aspartate oxidase  **NadB** , which synthesizes iminoaspartate as a substrate for  **NadA** , the next enzyme in the pathway.  Ref: 12496312 J Biol Chem, 2003
- Quinolinic acid is synthesized in E. coli by the enzymes L aspartate oxidase  [ **NADB** ]  and quinolinate synthase A, the genes of which are named  **nadB**  and  **nadA** .  Ref: 2187483 Biol Chem Hoppe Seyler, 1990

**Non-interactive relationship** (e.g. studied together, co-existance, homology, etc.)

- As NAD levels increase within the cell, the affinity of NadR for the operator regions of  **nadA** ,  **nadB** , and pncB increases, repressing the transcription of these target genes.  Ref: 2198247 J Bacteriol, 1990
- Using interrupted mating and transductional mapping procedures, three loci, designated  **nadA** ,  **nadB** , and nadC, were identified.  Ref: 203571 J Bacteriol, 1978
- Mutations at the nadI locus affect expression of the first two genes of NAD synthesis,  **nadA**  and  **nadB** , which are unlinked.  Ref: 3275606 J Bacteriol, 1988
- The nadI protein regulates transcription of the  **nadA**  and  **nadB**  biosynthetic genes and modulates activity of the NMN permease.  Ref: 1991724 J Bacteriol, 1991
- Common null mutations nadI show constitutive high level expression of the  **nadB**  and  **nadA**  genes.  Ref: 3275606 J Bacteriol, 1988
- The presence of cAMP cAMP receptor protein was essential for the complete derepression of  **nadA**  while no effect was evident upon  **nadB** .  Ref: 3934331 J Gen Microbiol, 1985
- High copy number vectors, carrying either gene  **nadA** ,  **nadB**  or  **nadA**   **nadB** , increased quinolinate production 1.5 fold, 2.0 fold and 15 fold respectively.  Ref: 2841129 Eur J Biochem, 1988
- The quinolinate synthetase A  [ **NADA** ]  protein was present in all nadC mutants and most but not all  **nadB**  mutants.  Ref: 238844 Eur J Biochem, 1975
- Evidence for the formation of an unstable intermediate in the synthesis of quinolinate from aspartate and dihydroxyacetone phosphate by Escherichia coli was obtained using toluenized cells of  **nadA**  and  **nadB**  mutants of this organism and partially purified A and B proteins in dialysis and membrane cone experiments.  Ref: 361684 J Bacteriol, 1978
- Increases in nadAB transcription directly correlated with decreases in intracellular NAD P levels, and kinetic studies indicated that the NAD analogue 6 amino NAD was ineffective in repressing either  **nadA**  or  **nadB** .  Ref: 3934331 J Gen Microbiol, 1985
- The separation of the  **nadB**  gene product from the  **nadA**  gene product and its purification.  Ref: 238844 Eur J Biochem, 1975
- Rare nadIs mutations cause constitutive low level expression of  **nadB**  and  **nadA** .  Ref: 3275606 J Bacteriol, 1988
- In Salmonella typhimurium, de novo synthesis of NAD is regulated through the transcriptional control of the  **nadA**  and  **nadB**  loci.  Ref: 2198247 J Bacteriol, 1990
- The arrangement of the loci on the Salmonella linkage map corresponded closely to the  **nadA** ,  **nadB** , and nadC loci on the Escherichia coli K 12 linkage map, indicating that the de novo pathway to nicotinamide adenine dinucleotide and the genes governing the enzymes involved in this pathway in S. typhimurium are very similar to those in E. coli.  Ref: 203571 J Bacteriol, 1978
- Transfer of cultures from aerobic to anaerobic conditions resulted in the partial derepression of both  **nadA**  and  **nadB** .  Ref: 3934331 J Gen Microbiol, 1985
- Cloning and characterization of quinolinate synthesis genes  **nadA**  and  **nadB** .  Ref: 2841129 Eur J Biochem, 1988
- ... all  **nadA**  and  **nadB**  mutants of S. typhimurium grew well in the presence of quinolinic acid.  Ref: 203571 J Bacteriol, 1978
- Finally, our data definitely exclude the possibility that other enzymes are involved in the biosynthesis of quinolinic acid in E. coli, since it is possible to synthesize quinolinic acid from l aspartate, dihydroxyacetone phosphate, and O 2 by using only  **nadA**  and  **nadB**  gene overexpressed products.  Ref: 10648170 Protein Expr Purif, 2000
- The  **nadA**  17 unit map position and  **nadB**  55 units genetic loci involved with quinolinic acid biosynthesis were both found to be regulated by the product of a nadR locus 99 units in a repression derepression manner while nadC 3 units expression appeared constitutive at the transcriptional level.  Ref: 3934331 J Gen Microbiol, 1985
- The nadI locus encodes a repressor protein acting on the unlinked  **nadA**  and  **nadB**  genes.  Ref: 3275606 J Bacteriol, 1988
- The two genes,  **nadA**  and  **nadB** , responsible for quinolinate biosynthesis from aspartate and dihydroxyacetone phosphate in Escherichia coli were cloned and characterized.  Ref: 2841129 Eur J Biochem, 1988
- A compound, isolated from the culture fluid of a nadC auxotroph of Escherichia coli grown in a minimal medium, supports the growth of both a  **nadA**  and a  **nadB**  mutant.  Ref: 163810 J Bacteriol, 1975
- The quinolinate synthetase B  [ **NADB** ]  protein exists in all  **nadA**  and nadC mutants examined.  Ref: 238844 Eur J Biochem, 1975
